# Development of Functional Fitness Prediction Equation in Korean Older Adults: The National Fitness Award 2015–2019

**DOI:** 10.3389/fphys.2022.896093

**Published:** 2022-05-10

**Authors:** Sung-Woo Kim, Hun-Young Park, Hoeryong Jung, Kiwon Lim

**Affiliations:** ^1^ Physical Activity and Performance Institute, Konkuk University, Seoul, South Korea; ^2^ Department of Sports Medicine and Science, Graduate School, Konkuk University, Seoul, South Korea; ^3^ Department of Mechanical Engineering, Konkuk University, Seoul, South Korea; ^4^ Department of Physical Education, Konkuk University, Seoul, South Korea

**Keywords:** older adults, functional fitness, multiple linear regression, hand grip strength, national fitness award

## Abstract

The main advantage of measuring functional fitness (FF) in older adults is that individual tests can estimate and track the rate of decline with age. This study aimed to develop a multiple linear regression model for predicting FF variables using easy-to-measure independent variables (e.g., sex, age, body mass index, and percent body fat) in Korean older adults. National Fitness Award datasets from the Republic of Korea were used in this analysis. The participants were aged ≥65 years and included 61,465 older men and 117,395 older women. The FF variables included the hand grip strength, lower body strength (30-s chair stand), lower body flexibility (chair sit-and-reach), coordination (figure of 8 walk), agility/dynamic balance (timed up-and-go), and aerobic endurance (2-min step test). An estimation multiple linear regression model was developed using a stepwise technique. In the regression model, the coefficient of determination in the hand grip strength test (adjusted R^2^ = 0.773, *p* < 0.001) was significantly high. However, the coefficient of determination in the 30-s chair stand (adjusted R^2^ = 0.296, *p* < 0.001), chair sit-and-reach (adjusted R^2^ = 0.435, *p* < 0.001), figure of 8 walk (adjusted R^2^ = 0.390, *p* < 0.001), timed up-and-go (adjusted R^2^ = 0.384, *p* < 0.001), and 2-min step tests (adjusted R^2^ = 0.196, *p* < 0.001) was significantly low to moderate. Our findings suggest that easy-to-measure independent variables can predict the hand grip strength in older adults. In future studies, explanatory power will be further improved if multiple linear regression analysis, including the physical activity level and nutritional status of older adults, is performed to predict the FF variables.

## Introduction

Recently, the worldwide population of individuals over 65 years of age has rapidly increased. Human aging is associated with gradual deterioration of various physiological systems, especially changes in the neuromuscular and cardiopulmonary systems (Hurst et al., 2019). Decreased cardiopulmonary function and muscle fitness are strongly associated with increased morbidity and mortality (Ruiz et al., 2008; Kodama et al., 2009). Improving and managing these physical components are the most effective strategies for reducing all causes and risks of cardiovascular death (Kim et al., 2018).

Lifestyle habits play an essential role in maintaining or promoting good health and quality of life, with physical activity and nutrition being the top priorities (Ignasiak et al., 2020). Previous studies have reported that physical inactivity decreases physiological aging parameters and causes limitations in daily mobility and functional ability, along with non-communicable diseases (Rikli R. E. and Jones C. J., 2013). The need for research and evaluation of the physical conditions of older adults stems from various environmental factors (e.g., different living, cultural, ethnic, genetic, and geographical conditions) and socioeconomic changes (Ignasiak et al., 2018). Most research conducted on older adults is related to the role of physical activity in the prevention and treatment of medical problems and diseases (Ignasiak et al., 2020). In addition to these studies, it is important to track the biological aging process of older adults, whose physical ability to live independently is important.

Functional fitness (FF) is defined as an individual’s ability to perform daily life activities without difficulties and includes muscle strength, flexibility, agility/dynamic balance, and aerobic endurance (Rikli R. E. and Jones C. J., 2013). It has been reported as an independent factor with the most significant influence on mortality (Church et al., 2007). In elderly individuals, the hand grip strength (HGS) is an essential indicator of muscle strength, and a decrease in this variable is closely related to an increase in the risk of falls (Savino et al., 2013; Cruz-Jentoft et al., 2014). In addition, the walking speed is used as an indicator of the quality of neuromuscular function and is a major factor in determining healthy aging (Larsson et al., 2019; Perez-Sousa et al., 2019). These physical abilities are also used to diagnose dysfunction and evaluate independence in older adults (Graham et al., 2010; López-Teros et al., 2014; Cruz-Jentoft et al., 2019). The main advantage of measuring FF in older adults is that individual tests can estimate and track the rate of decline with age (Rikli and Jones, 1999). Although it is expected that FF variables decrease with aging, the level and ratio of this decrease can indicate the threat of loss of functional independence (Ignasiak et al., 2020). Therefore, it is necessary to monitor and evaluate the physical abilities of older adults to determine the appropriate programs and treatment methods. However, older adults with mobility restrictions, such as osteoporosis and sarcopenia, have difficulty performing the FF tests. Additionally, laboratory methods can accurately measure the FF variables. However, they may not be feasible for the entire population owing to cost, time constraints, and the need for qualified technicians and sophisticated devices (Kim et al., 2021). Recently, many convenient products for the healthcare of older adults have been developed.

Therefore, our study aimed to develop a multiple linear regression model for predicting FF variables (e.g., HGS, lower body strength, lower body flexibility, coordination, agility/dynamic balance, and aerobic endurance) using easy-to-measure independent variables [e.g., sex, age, body mass index (BMI), and percent body fat] in Korean older adults.

## Materials and Methods

### Datasets

National Fitness Award (NFA) datasets from the Republic of Korea were used in this analysis. The NFA is a nationwide test conducted at 75 sites that assesses the physical fitness of the general population in the Republic of Korea. In the NFA dataset, data on independent and FF variables of the older adults were provided, but physical activity, nutrition, chronic diseases status, and hemodynamic data were not provided. This study included older adults (age: ≥65 years) who participated in the NFA between 2015 and 2019. Among a total of 210,490 adults, we excluded participants who had no data on their independent (*n* = 44) and FF variables (*n* = 31,486). Finally, 178,960 adults (older men: *n* = 61,465, older women: *n* = 117,395) were included in the analysis. The older adults were divided at a ratio of 7:3 in the Bernoulli trial. Approximately 70% of the divided data (older adults: *n* = 125,272, older men: *n* = 42,991, older women: *n* = 82,281) were used to develop the FF estimation formula based on sex, age, BMI, and percent body fat, and approximately 30% of the data (older adults: *n* = 53,688, older men: *n* = 18,474, older women: *n* = 35,214) were used to perform the validity test. The power test was performed using G*Power 3.1.9.2 (Franz Faul, University of Kiel, Kiel, Germany) at the tails of two; the following parameters were used for all statistical tests: H1 ρ^2^ = 0.01, H0 ρ^2^ = 0, significance level = 0.05 (α = 0.05), power = 0.9, and number of predictors = 4. G*Power showed that 1769 participants were needed to achieve sufficient power for this study. The study was conducted in accordance with the guidelines of the Declaration of Helsinki and was approved by the Institutional Review Board of Konkuk University (7001355-202101-E-132). All participants provided informed consent prior to enrollment. The population characteristics are listed in Table 1.

**TABLE 1:** Characteristics of the study population.

Variables	Regression model data			Validity test data		
	Older adults (*n* = 125,272)	Older men (*n* = 42,991)	Older women (*n* = 82,281)	Older adults (*n* = 53,688)	Older men (*n* = 18,474)	Older women (*n* = 35,214)
Age (years)	72.79 ± 5.57	73.26 ± 5.45	72.55 ± 5.62	72.78 ± 5.55	73.31 ± 5.45	72.51 ± 5.59
Height (cm)	156.74 ± 8.27	165.10 ± 5.86	152.37 ± 5.54	156.76 ± 8.25	165.10 ± 5.79	152.38 ± 5.52
Weight (kg)	60.56 ± 9.30	66.33 ± 8.87	57.54 ± 8.00	60.56 ± 9.27	66.39 ± 8.84	57.49 ± 7.93
BMI (kg/m^2^)	24.61 ± 3.03	24.31 ± 2.80	24.77 ± 3.13	24.61 ± 3.00	24.33 ± 2.79	24.75 ± 3.09
Percent body fat (%)	31.90 ± 7.69	26.01 ± 6.39	34.97 ± 6.42	31.88 ± 7.66	26.06 ± 6.42	34.93 ± 6.38
HGS (kg)	23.33 ± 7.71	30.76 ± 6.66	19.45 ± 4.84	23.37 ± 7.69	30.75 ± 6.65	19.49 ± 4.82
30-s chair stand (n)	19.03 ± 6.27	20.58 ± 6.40	18.23 ± 6.05	19.05 ± 6.26	20.53 ± 6.40	18.27 ± 6.05
Chair sit-and-reach (cm)	9.91 ± 9.70	3.87 ± 9.70	13.06 ± 8.06	9.91 ± 9.69	3.79 ± 9.64	13.12 ± 8.02
Figure of 8 walk (sec)	27.32 ± 7.70	26.04 ± 7.01	28.00 ± 7.96	27.31 ± 7.70	26.13 ± 7.16	27.95 ± 7.90
Timed up-and-go (sec)	6.59 ± 2.00	6.20 ± 1.81	6.79 ± 2.07	6.58 ± 1.96	6.20 ± 1.78	6.77 ± 2.02
2-min step test (*n*)	102.69 ± 26.83	107.20 ± 24.90	100.31 ± 27.49	102.81 ± 26.46	107.02 ± 24.50	100.58 ± 27.18

Note: Values are expressed as mean ± SD. BMI, body mass index; HGS, hand grip strength.

### Measurement of Independent Variable

Height was measured to the nearest 0.1 cm using a stadiometer (BSM 370, Inbody, Seoul, South Korea). Weight and percent body fat were measured using bioelectrical impedance analysis (Inbody 770, Inbody). BMI was calculated by dividing the weight (kg) by the height squared (m^2^).

### FF Variables

All the FF variables were measured by certified health and physical fitness instructors. These variables included the HGS, lower body strength (30-s chair stand), lower body flexibility (chair sit-and-reach), coordination (figure of 8 walk), agility/dynamic balance (timed up-and-go), and aerobic endurance (2-min step test). The descriptions of the tests were as follows.

### HGS Test (kg)

Isometric muscle strength was assessed using a hand dynamometer (GRIP-D 5101, Takei, Niigata, Japan). The participants held the dynamometer with their preferred hand and squeezed it as forcefully as possible. All the participants were tested twice, and the best result was recorded to the nearest 0.1 kg.

### Thirty-Second Chair Stand Test (Number of Times)

The participants were instructed to sit upright on a chair with their hands crossed and placed on their chest. The number of times they could stand and sit within 30 s after the start of the signal was measured.

### Chair Sit-and-Reach Test (cm)

From a sitting position on the edge of a chair with one leg extended and the hands reaching toward the toes, the distance (cm) (+ or −) between the extended fingers and tip of the toe was measured. The scores were recorded to the nearest 0.1 cm.

### Figure of 8 Walk Test (s)

A 3.6-m wide × 1.6-m long rectangular line was drawn on the floor, a cone hat was fixed in both corners, and a chair was placed 2.4 m away from the cone. The participants were instructed to sit on the chair in the center of the corner in front of the square and on the cone-shaped chair in the back right according to the slogan “Start.” Without a break, they got up from the chair again, returned to the left cone on the back, and sat on the chair. This process was repeated twice, and the time required within 0.1 s was measured. The tests were performed after one or two sessions.

### Timed Up-and-Go Test (s)

The participants sat on a chair and leaned back against the wall. They were instructed to get up from the chair, walk toward a cone placed 3 m away, turn around the cone, return to the chair, and sit down again as rapidly as possible without running. The time required to complete the activity was recorded.

### Two-Minute Step Test (Number of Times)

The participants were instructed to step in place repeatedly for 2 min by raising each knee midway between the patella and iliac crest. A score was assigned on the basis of the number of times the right knee reached the required level.

### Statistical Analysis

Means and standard deviations were calculated for all measured variables. The normality of the distribution of all outcome variables was verified using the Kolmogorov–Smirnov test. To perform multiple linear regression analysis, we used the β value (regression coefficient) to confirm whether the independent variables had explanatory power. We used a stepwise regression analysis model, which is indicated when multiple independent variables are considered as predictors. The stepwise regression technique maximizes the estimated power with a minimum number of independent variables. Multiple linear regression analysis with the stepwise technique was then performed to predict the FF variables (HGS, lower body strength, lower body flexibility, coordination, agility/dynamic balance, and aerobic endurance) using independent variables (sex, age, BMI, and percent body fat). In addition, we rigorously conformed to the basic assumptions of the regression model: linearity, independence, autocorrelation, homoscedasticity, continuity, normality, and outliers. Outlier data in the multiple linear regression model were identified and removed when the absolute value of the studentized residual (SRE) was ≥2. The validity of the regression model was tested using approximately 30% of the total data, which had already been divided in the Bernoulli trial. This was not included in the regression model. The validation test calculated the predicted values of the FF variables using the regression equation, and the mean error and standard error of estimation (SEE) were calculated using Formula 1, Formula 2. A two-tailed Pearson correlation analysis was performed between the independent and FF variables and estimated the relationships between the measured and predicted FF variables. The Statistical Package for the Social Sciences version 26.0 (IBM Corporation, Armonk, NY, United States) was used for the analysis, and the level of significance was set at 0.05.

$$\mathrm{Mean}\;\mathrm{error}\left(\%\right)=\frac{\sum \frac{\mathrm{Measured}\;\mathrm{value}-\mathrm{Predicted}\;\mathrm{value}}{\mathrm{Measured}\;\mathrm{value}}*100}{N}$$

Formula 1 : Calculation formula for the mean error.

$$\mathrm{Standard}\;\mathrm{errors}\;\mathrm{of}\;\mathrm{estimation}=\frac{\sum {\left(\mathrm{Mesured}\;\mathrm{value}-\mathrm{Predicted}\;\mathrm{value}\right)}^{2}}{N-2}$$

Formula 2 : Calculation formula for the standard error of estimation.

## Results

For each developed multiple linear regression model, an F-test was used to validate the significance of the model. The multiple regression analyses revealed that the regression coefficients for the selected independent variables were significant. The multiple regression analyses for each model included the coefficients of determination (R^2^), adjusted coefficients of determination (adjusted R^2^), and SEE. The correlations between the independent and FF variables are shown in Table 2.

**TABLE 2:** Correlation coefficients between independent variables and FF variables for the estimating regression model.

Independent variables	Sex	Age	BMI	Percent body fat
FF variables				
HGS	−0.697**	−0.221**	0.015**	−0.468**
30-s chair stand	−0.176**	−0.316**	−0.116**	−0.248**
Chair sit-and-reach	0.452**	−0.245**	−0.027**	0.149**
Figure of 8 walk	0.118**	0.430**	0.109**	0.208**
Timed up-and-go	0.140**	0.407**	0.107**	0.208**
2-min step test	−0.120**	−0.313**	−0.080**	−0.185**

Note: Significant correlation between measured FF variables and independent variables, **p < 0.01. FF, functional fitness; BMI, body mass index; HGS, hand grip strength.

### Performance Evaluation of the Regression Models and Equations

The detailed results of the multiple regression analysis using the FF variables are presented in Table 3. The estimated explanatory power of the HGS test regression model was 58.5%, and the SEE was 4.97 kg (*F* = 43,915.138, *p* < 0.001). The explanatory power of the 30-s chair stand test regression model was 16.3%, and the SEE was 5.74 n (*F* = 6,074.535, *p* < 0.001). The explanatory power of the chair sit-and-reach test regression model was 26.1%, and the SEE was 8.34 cm (*F* = 10,989.238, *p* < 0.001). The explanatory power of the estimated figure of 8 walk test regression model was 22.8%, and the SEE was 6.77 s (*F* = 7,621.883, *p* < 0.001). The explanatory power of the estimated timed up-and-go test regression model was 21.0%, and the SEE was 1.78 s (*F* = 8,289.869, *p* < 0.001). Finally, the explanatory power of the estimated 2-min step test regression model was 13.1%, and the SEE was 25.00 n (*F* = 6,002.718, *p* < 0.001).

**TABLE 3:** Estimated regression equations predicting FF variables.

Regression model	R	R^2^	Adjusted R^2^	*F*-value	*p*-value	SEE
HGS = 58.625 − (9.421 * sex) − (0.334 * age) − (0.266 * percent body fat) + (0.533 * BMI)	0.765	0.585	0.585	43,915.138	0.000	4.97 kg
30-s chair stand = 52.615 − (0.359 * age) − (0.141 * percent body fat) − (1.337 * sex) − (0.031 * BMI)	0.404	0.163	0.163	6074.535	0.000	5.74 n
Chair sit-and-reach = 23.613 + (10.428 * sex) − (0.363 * age) − (0.168 * percent body fat) + (0.033 * BMI)	0.511	0.261	0.261	10,989.238	0.000	8.34 cm
Figure of 8 walk = −24.570 + (0.587 * age) + (0.119 * percent body fat) + (1.274 * sex) + (0.116 * BMI)	0.478	0.228	0.228	7621.883	0.000	6.77 s
Timed up-and-go = −6.656 + (0.149 * age) + (0.027 * percent body fat) + (0.444 * sex) + (0.034 * BMI)	0.459	0.210	0.210	8289.869	0.000	1.78 s
2-min step test = 233.873 − (1.510 * age) − (0.477 * percent body fat) − (3.661 * sex)	0.362	0.131	0.131	6002.718	0.000	25.00 n

Note. FF, functional fitness; SEE, standard error of estimation; sex, 1 = men, 2 = women; BMI, body mass index; HGS, hand grip strength.

### Performance Evaluation of the Regression Models and Equations Without Outlier Data

Table 4 shows the results of the multiple regression analysis using the FF variables without outlier data. The explanatory power of the HGS test regression model (SRE 32: *n* = 101,438) was 77.3%, and the SEE was 3.12 kg (*F* = 86,217.720, *p* < 0.001). The explanatory power of the developed 30-s chair stand test regression model (SRE 39: *n* = 102,726) was 29.6%, and the SEE was 3.83 n (*F* = 10,822.040, *p* < 0.001). The explanatory power of the chair sit-and-reach test regression model (SRE 35: *n* = 102,640) was 43.5%, and the SEE was 5.43 cm (*F* = 26,392.462, *p* < 0.001). The explanatory power of the estimated figure of 8 walk test regression model (SRE 22: *n* = 79,724) was 39.0%, and the SEE was 3.11 s (*F* = 12,742.839, *p* < 0.001). The explanatory power of the estimated timed up-and-go test regression model (SRE 36: *n* = 94,621) was 38.4%, and the SEE was 0.70 s (*F* = 14,752.920, *p* < 0.001). Finally, the explanatory power of the estimated 2-min step test regression model (SRE 28: *n* = 91,420) was 19.6%, and the SEE was 12.47 n (*F* = 7,441.515, *p* < 0.001).

**TABLE 4:** Estimated regression equations predicting FF variables without outlier data.

Regression model	R	R^2^	Adjusted R^2^	*F*-value	*p*-value	SEE
HGS (SRE 32: *n* = 101,438) = 57.708 − (9.602 * sex) − (0.315 * age) − (0.256 * percent body fat) + (0.528 * BMI)	0.879	0.773	0.773	86,217.720	0.000	3.12 kg
30-s chair stand (SRE 39: *n* = 102,726) = 50.825 − (0.349 * age) − (0.132 * percent body fat) − (1.481 * sex) − (0.025 * BMI)	0.544	0.296	0.296	10,822.040	0.000	3.83 n
Chair sit-and-reach (SRE 35: *n* = 102,640) = 27.687 + (10.064 * sex) − (0.384 * age) − (0.162 * percent body fat)	0.660	0.435	0.435	26,392.462	0.000	5.43 cm
Figure of 8 walk (SRE 22: *n* = 79,724) = −12.442 + (0.402 * age) + (0.076 * percent body fat) + (1.309 * sex) + (0.156 * BMI)	0.625	0.390	0.390	12,742.839	0.000	3.11 s
Timed up-and-go (SRE 36: *n* = 94,621) = −2.698 + (0.091 * age) + (0.015 * percent body fat) + (0.402 * sex) + (0.038 * BMI)	0.620	0.384	0.384	14,752.920	0.000	0.70 s
2-min step test (SRE 28: *n* = 91,420) = 194.029 − (1.008 * age) − (0.305 * percent body fat) − (2.313 * sex)	0.443	0.196	0.196	7441.515	0.000	12.47 n

Note. FF, functional fitness; SEE, standard error of estimation; sex, 1 = men, 2 = women; BMI, body mass index; HGS, hand grip strength.

### Regression Model Validity

The validity of the developed regression models was calculated using data not included in the multiple regression analyses. In all regression models of the FF variables, the mean error ranged from −24.50% to 4.00% (HGS test: −8.05%, 30-s chair stand test: −7.68%, chair sit-and-reach test: −0.58%, figure of 8 walk test: 1.90%, timed up-and-go test: 4.00%, and 2-min step test: −24.50%), and the SEE was higher than that of the developed regression model (Table 5).

**TABLE 5:** Validity of estimating the regression model.

	HGS	30-s chair stand	Chair sit-and-reach	Figure of 8 walk	Timed up-and-go	2-min step test
Mean error (%)	−8.05	−7.68	−0.58	1.90	4.00	−24.50
SEE	4.96 kg	5.81 n	8.40 cm	7.11 s	1.86 s	25.25 n

Note. SEE, standard error of estimation; HGS, hand grip strength.

### Relationship Between the Measured and Predicted FF Variables

**FIGURE 1:**
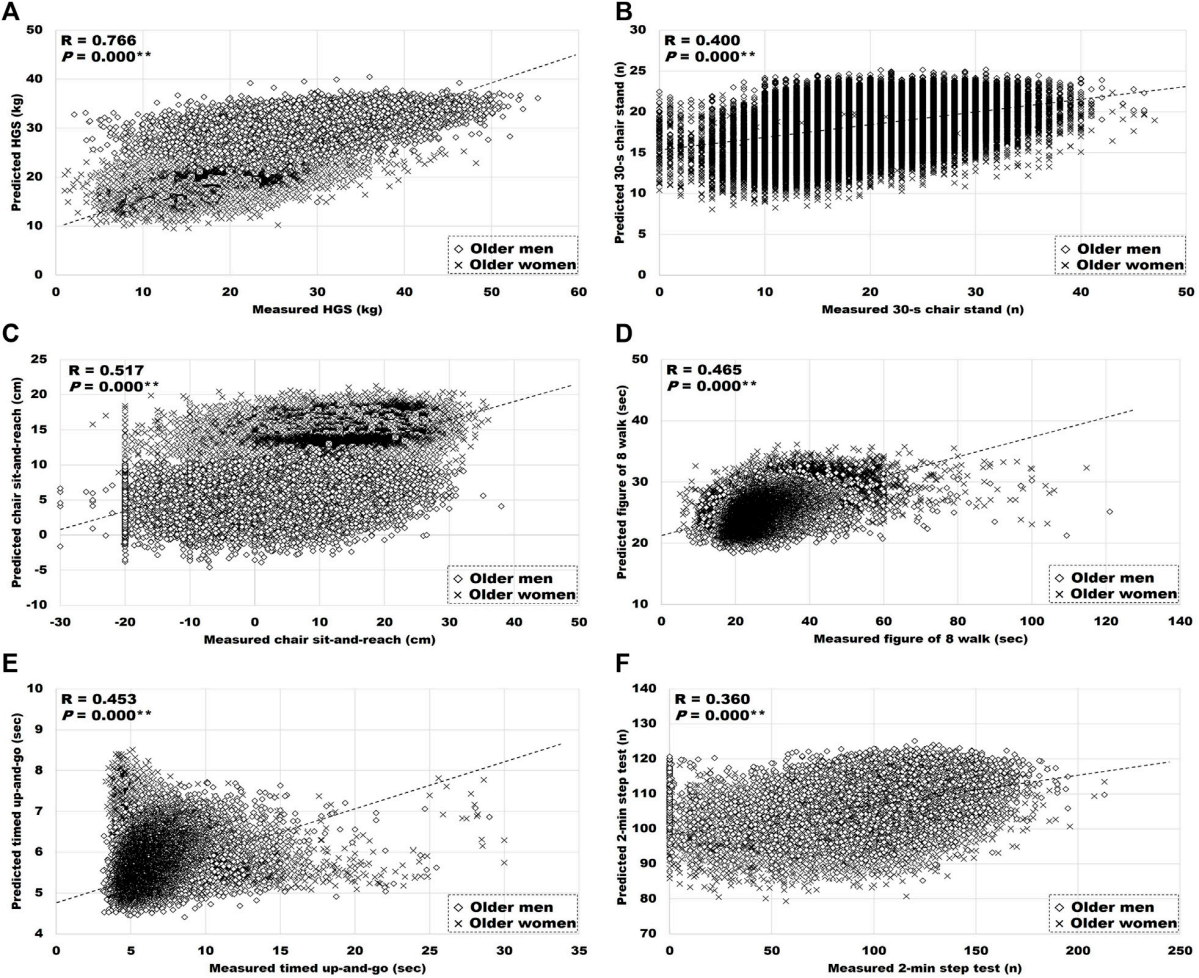
Relationship between measured, and predicted FF. **(A)** HGS: hand grip strength, **(B)** 30-s chair stand, **(C)** chair sit-and-reach, **(D)** figure of 8 walk, **(E)** timed up-and-go, and **(F)** 2-min step test. Significant correlation between measured and predicted variables, **p < 0.01.

Figure 1 presents the relationship between the measured and predicted FF variables. The measured FF variables were positively correlated with the predicted HGS (r = 0.766, *p* < 0.01) and 30-s chair stand (r = 0.400, *p* < 0.01), chair sit-and-reach (r = 0.517, *p* < 0.01), figure of 8 walk (r = 0.465, *p* < 0.01), timed up-and-go (r = 0.453, *p* < 0.01), and 2-min step test results (r = 0.360, *p* < 0.01).

## Discussion

Many researchers have conducted studies to assess health conditions using FF while assuming that FF variables are a reliable healthcare index in older adults. In terms of healthcare, it would be helpful to develop tools that can measure and evaluate FF in daily life. In previous studies, most of the FF variables in older adults were compared with FF measurement parameters developed or regression equations targeting relatively small numbers of samples (Mukherjee et al., 2020; Pan et al., 2020; Yee et al., 2021). Herein, we aimed to develop a multiple linear regression model for estimating FF variables in Korean older adults using easy-to-measure independent variables. It is important to remove outliers before developing multiple linear regression models to estimate FF variables. This is because outliers increase the prediction errors. In our study, the absolute value criterion of the SRE was used to eliminate the outliers. In the developed multiple linear regression model, the coefficient of determination of the FF variable was high only in the HGS test. The mean explanatory power of the HGS test regression model was 77.3%.

The HGS is a simple measure for public health research and clinical practice (Mehmet et al., 2020), which evaluates the function of a single muscle group (Buehring et al., 2015). It has also been used to evaluate generalized effects on the musculoskeletal system in older adults, such as stroke, fatigue, and obesity (Mehmet et al., 2020). The European Working Party on Sarcopenia in Older People recommends the HGS as the most common practical measure of muscle strength available in clinical practice (Cruz-Jentoft et al., 2010). The HGS has been used to evaluate various anthropometric factors (Mukherjee et al., 2020). In our study, the mean explanatory power of the HGS test regression model [57.708 − (9.602 × sex_male = 1; female = 2_) + (0.315 × age) − (0.256 × percent body fat) − (0.528 × BMI)] was 77.3% (adjusted R^2^). In a previous study, 14 anthropometric dimension variables (i.e., weight, stature, eye, mid-shoulder, acromion, cervical, mid-shoulder, elbow rest, knee height, popliteal, hand length, palm length, hand breadth with thumb, and grip inside diameter maximum) predicted 52.25% (adjusted R^2^) of the HGS of elderly individuals (*n* = 38) in India (Mukherjee et al., 2020). In another study, the independent variables of height, sex, age, exercise time, weight, waist circumference, diabetes mellitus, heart disease, and living status accounted for 53.3% (R^2^) of the variance of the HGS in elderly individuals (age: 65 years and older; total: *n* = 2,470; men: *n* = 998; women: *n* = 1,472) in Taiwan (Pan et al., 2020). Our study confirmed that the multiple linear regression model formulation was more accurate and straightforward than the predictive power of previous studies.

It is necessary to maintain FF in older adults to prevent mobility disorders associated with aging. Muscle strength is a key variable in FF, along with flexibility, dynamic balance, and aerobic endurance. Similar to our study, previous studies have been conducted to predict muscle strength among FF variables in older adults. In one study, 42.6% (adjusted R^2^) of the variability in the 30-s chair stand test result could be explained by the timed up-and-go and 6-min walk test results, HGS, gait speed, fat mass index, weight, height, age, and sex in community-dwelling older adults (age: 67.3 ± 7.0 years; overall: *n* = 887; men: *n* = 240; women: *n* = 647) (Yee et al., 2021). In our study, the estimated mean explanatory power of the 30-s chair stand test multiple linear regression model [50.825 − (0.349 × age) − (0.132 × percent body fat) − (1.481 × sex_male=1; female=2_) − (0.025 × BMI)] was 29.6% (adjusted R^2^). The predictive power of the linear regression model was higher in previous studies than in our study; however, both had a low power when statistical criteria were used for the evaluation. Our study used multiple linear regression to analyze four independent variables that were easy to measure; however, some FF variables were included in previous studies. In our multiple linear regression models of FF variables, the coefficient of determination in the 30-s chair stand (adjusted R^2^ = 0.296), chair sit-and-reach (adjusted R^2^ = 0.435), figure of 8 walk (adjusted R^2^ = 0.390), timed up-and-go (adjusted R^2^ = 0.384), and 2-min step tests (adjusted R^2^ = 0.196) was significantly low to mid-range. Rikli and Jones developed and improved senior fitness tests that can evaluate the FF of older adults to meet safety conditions, ease of performance, reliability, repeatability between 0.80 and 0.97, and accuracy between 0.79 and 0.97 (Rikli and Jones, 1999; Rikli R. E. and Jones C. J., 2013; Rikli R. E. and Jones C. J., 2013). Our study aimed to predict FF variables using only independent variables that were easy to measure; however, the coefficient of determination was low, which is insufficient for use in clinical practice and healthcare.

## Limitations

This study has some limitations. The role of FF and nutrition in reducing the progression of aging is becoming increasingly critical (Clegg and Williams, 2018). FF is defined as the essential ability to live independently (Rikli R. E. and Jones C. J., 2013), and nutrition is well known as a major modifiable behavior (Clegg and Williams, 2018). Previous studies have reported that improving FF and nutritional factors leads to a healthy and independent aging process by preventing functional aging-related limitations (Clegg and Williams, 2018; Hurst et al., 2019). In addition, the daily activities of older adults decrease with aging, although it is well known that physical activity is important for preventing chronic health problems (Goldspink, 2005), living independently (Westerterp, 2000), and improving quality of life (Brill, 2004). A significant association between physical activity and the current functional status of older women has been reported (Brach et al., 2003). Therefore, physical activity plays an important role in maintaining FF (Milanović et al., 2013). Also, various diseases occur in the older adults due to physiological aging (Ruiz et al., 2008; Kodama et al., 2009; Hurst et al., 2019). However, the relationship with the FF variables could not be analyzed herein because there was no information on the disease status, physical activity level, and nutrition in the NFA dataset. Further studies should establish models for predicting FF variables based on chronic diseases and hemodynamic status in older adults.

## Conclusion

This study demonstrated that the variability of the HGS alone in older adults could be explained by sex, age, BMI, and percent body fat. It is difficult to predict the FF variables in older adults using only four independent variables that are easy to measure. In future studies, the explanatory power will be further improved if multiple linear regression analysis, including the physical activity level and nutritional status of older adults, is performed to predict the FF variables.

## Data Availability

The original contributions presented in the study are included in the article/Supplementary Materials, further inquiries can be directed to the corresponding author.
